# Patient and surgeon predictors of achieving the critical view of safety in laparoscopic cholecystectomy: a prospective cohort study

**DOI:** 10.3389/fsurg.2025.1661510

**Published:** 2025-09-25

**Authors:** Rami Addasi, Lana Al-Sabe’, Kareem AlRawabdeh, Rand Abu-Zayed, Abdallah Alaarag, Marcelo A. F. Ribeiro, Ahmed H. Helmy, M. S. El Muhtaseb, Salam Daradkeh

**Affiliations:** 1 Department of General Surgery, School of Medicine, The University of Jordan, Amman, Jordan; 2 School of Medicine, The University of Jordan, Amman, Jordan; 3 HCA Florida Healthcare/USF Morsani College of Medicine, Citrus Hospital, Inverness, FL, United States; 4 Department of Surgery, University of Maryland—R Adams Cowley Shock Trauma Center, Baltimore, MD, United States; 5 Department of General Surgery, Theodor Bilharze Research Institute, Giza, Egypt

**Keywords:** laparoscopic cholecystectomy, critical view of safety, Calot's triangle, bile duct injury, intraoperative cholangiography, Nassar difficulty grading scale, Tokyo guidelines, acute cholecystitis

## Abstract

**Introduction:**

The Critical View of Safety (CVS) is a cornerstone of safe laparoscopic cholecystectomy (LC), aimed at minimizing the risk of bile duct injury (BDI). However, consistent achievement of CVS remains a challenge in surgical practice. The primary outcome of this study was to assess the rate of CVS achievement and to identify patient, disease and surgeon related predictors.

**Methods:**

A prospective cohort of 150 patients undergoing LC was analyzed. Demographic data, preoperative risk factors, intraoperative variables, and surgeon characteristics were examined. CVS assessment was performed using Strasberg's criteria. Binary logistic regression and Chi-squared test were used to identify independent predictors of CVS achievement.

**Results:**

The rate of CVS achievement in this study was 69.6% among consultants and 60.0% among residents. Logistic regression identified ASA grade I (*p* = 0.031), emergency surgery (*p* = 0.01), acute cholecystitis (*p* = 0.031), and non-HPB surgeons (*p* < 0.001) were associated with higher rate of CVS achievement. The higher rate of CVS achievement among non-HPB surgeons may reflect differences in case complexity, documentation practices & stricter adherence to protocols. Other factors including level of surgeon experience, Tokyo severity grade, intraoperative Nassar difficulty grading scale, age, male gender, BMI, diabetes mellitus and clinical frailty score were not significant. There were zero cases of bile duct injury in this study, precluding analysis of CVS failure impact on BDI.

**Conclusion:**

Both preoperative and intraoperative factors can influence a surgeon's ability to achieve CVS. In our study, lower ASA grade, emergency cholecystectomies, acute cholecystitis and operations performed by non-HPB surgeons were associated with a higher likelihood of achieving CVS. Standardized protocols and structured training may help improve CVS documentation across practice settings.

## 1 Introduction

Laparoscopic cholecystectomy (LC) was first performed by Erich Mühe in 1985 (1), and it quickly became the gold standard operation for patients with symptomatic gallstones (2, 3).

Despite its clear benefits, including faster recovery and decreased postoperative pain (4), LC has been associated with increased bile duct injury (BDI) (5). Major BDI rates remain in the range of 0.3%–0.5% for LC (6) and has remained largely unchanged over time despite increasing experience with the procedure and employing better training, imaging modalities and strategic operative concepts, such as Strasberg's Critical View of Safety (CVS) (7, 8). The misinterpretation of anatomical landmarks is the primary cause of intraoperative biliary and vascular injuries (9).

The Critical View of Safety (CVS) technique was introduced in 1995 by Strasberg et al. (5) to prevent BDI. Despite universal endorsement, consistent achievement of CVS varies and may be influenced by multiple factors including patient physiology, disease severity, and surgeon characteristics (10). However, there is limited prospective data on surgeon-related factors such as experience and subspecialty.

The study aimed to prospectively assess the rate of CVS achievement and to identify patient-related, disease-related and surgeon-related factors associated with the successful achievement of CVS. Secondary outcomes were to evaluate the incidence of BDI during LC and to investigate the potential association between failure to achieve CVS and 30-day postoperative outcomes.

While prior studies have explored potential factors influencing CVS achievement, few studies have prospectively assessed the influence of patient, disease, and surgeon related factors. This study aimed to fill this gap.

## 2 Methods

### 2.1 Study design

A prospective analysis was performed on all consecutive patients (*n* = 150) who underwent laparoscopic cholecystectomy at Jordan University Hospital between July 2023 and November 2023. Informed written consent was obtained from all participants prior to enrollment.

The inclusion criteria was all adult patients (aged ≥18 years) who underwent elective and emergency laparoscopic cholecystectomies. Exclusion criteria included patients undergoing cholecystectomy as a part of another abdominal surgery.

CVS was considered achieved only if all three Strasberg criteria were fulfilled. CVS assessment was based solely on surgeon documentation in operative notes without objective video review introducing a potential risk of documentation bias.

Approval was obtained from the institutional review board committee at Jordan University hospital prior to data collection.

### 2.2 Data collection

Demographic and clinical data were collected, including age, gender, BMI, ASA grade, clinical frailty scale, comorbidities, indication for surgery imaging findings, and Tokyo severity grade (8). Operative details included intraoperative Nassar difficulty grading scale (9) and surgeon experience. Patients were classified into two groups as the CVS and the no CVS groups. All surgeries were performed at a single academic center by surgeons with varying subspecialties.

### 2.3 Statistical analysis

Data were analyzed using SPSS Statistics v24.0 (IBM, Armonk, NY, USA). Descriptive statistics, statistics were used to summarize patient characteristics, whereas mean and standard deviation were used for continuous variables.

The chi-square test and Fisher's exact test were employed to assess associations between categorical variables and CVS achievement.

Univariate and multivariable logistic regression were performed to evaluate predictors of CVS achievement. Significance was set at *p* < 0.05. Chi-square testing was used for secondary outcomes related to postoperative complications.

## 3 Results

### 3.1 Baseline characteristics

Of 150 patients who underwent laparoscopic cholecystectomy, 104 (69.3%) were female and 46 (30.7%) were male, with a mean age of 50 (19–81) years.

The most common ASA grade was II (56.7%). Pre-obesity (25.0–29.0) was the most common BMI and most patients were classified as “Well” on the clinical frailty scale. (*n* = 66, 44%) (Table 1).

**Table 1:** Patient characteristics.

Variable		Frequency	Percentage
Age	18–39	53	35.3%
	40–59	70	46.7%
	60–81	27	18.0%
Sex	Female	104	69.3%
	Male	46	30.7%
ASA grade	I (normally healthy patient)	47	31.3%
	II (mild systemic disease without significant functional limitation)	85	56.7%
	III (severe systemic disease with significant functional limitation)	17	11.3%
	IV (severe systemic disease with constant threat to life)	1	0.7%
Body mass index (BMI)	Underweight BMI Below 18.5	0	0.0%
	Normal weight BMI 18.5–24.9	33	22.0%
	Pre-obesity BMI 25.0–29.9	55	36.7%
	Obesity class I BMI 30.0–34.9	37	24.7%
	Obesity class II BMI 35.0–39.9	16	10.7%
	Obesity class III BMI 40+	6	4.0%
	unknown	3	2.0%
Clinical frailty scale	Very fit	28	18.7%
	Well	66	44.0%
	Managing well	44	29.3%
	Vulnerable	8	5.3%
	Mildly frail	3	2.0%
	Moderately frail	1	0.7%

Most of the patients this study had no comorbidities (*n* = 95, 63.3%). The most common comorbidities were HTN and Diabetes Mellitus.

### 3.2 Indications for surgery and preoperative imaging

**Figure 1:**
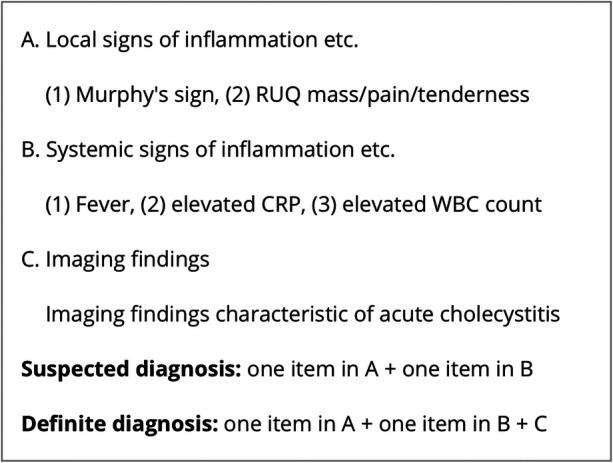
Tg18 diagnostic criteria for acute cholecystitis (11).

Biliary colic (*n* = 86, 57.3%), acute cholecystitis (*n* = 50, 33.3%) and biliary pancreatitis (*n* = 7, 4.7%) were the most frequent indications for LC. Other indications included gallbladder polyp, gallbladder dyskinesia, common bile duct stones, and chronic cholecystitis (Table 2). Acute cholecystitis was diagnosed according to the criteria outlined in the 2018 Tokyo Guidelines (Figure 1) (11).

**Table 2:** Indications for surgery.

Variable	Acute cholecystitis *N* = 50, (33.3%)	Biliary colic *N* = 86, (57.3%)	Biliary pancreatitis *N* = 7, (4.7%)	Other *N* = 7, (4.7%)
Imagine findings				
gallstones *N* (%)	49 (98%)	85 (98.8%)	7 (100%)	2 (28.6%)
Thick-walled gallbladder *N* (%)	23 (46%)	9 (10.5%)	1 (14.3%)	5 (71.4%)
Pericholecystic fluid *N* (%)	25 (50%)	3 (3.5%)	3 (42.9%)	0
CBD stones *N* (%)	2 (4%)	1 (1.2%)	2 (28.6%)	1 (14.3%)
Dilated CBD *N* (%)	3 (6%)	0	1 (14.3%)	1 (14.3%)
Urgency of surgery				
elective	22 (44%)	85 (98.8%)	4 (57.1%)	7 (100%)
Emergency	28 (56%)	1 (1.2%)	3 (42.9%)	0

Other, gallbladder polyps, gallbladder dyskinesia, common bile duct stones, and chronic cholecystitis.

All patients in this study underwent abdominal ultrasonography. Gallstones were identified in 95.3% of patients (*n* = 143). Among those diagnosed with acute cholecystitis, 50% had pericholecystic fluid (*n* = 25), while gallbladder wall thickening was present in 46% (*n* = 23). Emergency surgery was performed in 56% of patients with acute cholecystitis (*n* = 28), whereas 98.8% of biliary colic patients (*n* = 85) underwent elective surgery, with only 1.2% (*n* = 1) requiring emergency surgery (Table 2).

### 3.3 Operative details

All procedures were completed laparoscopically, with no conversions to open surgery. Intraoperative cholangiography (IOC) was not performed in any case. IOC is not routinely performed at our institution, as there is no evidence-based consensus that its routine use significantly reduces the risk of BDI to justify its widespread application (12).

A total of seven consultants participated in the study, including three hepatopancreatobiliary (HPB) surgeons and four from other subspecialties: colorectal, oncology, minimally invasive, and oesophagogastric. All consultants had performed more than 200 cholecystectomies, while all residents had performed fewer than 50 cholecystectomies at the time of the study.

**Figure 2:**
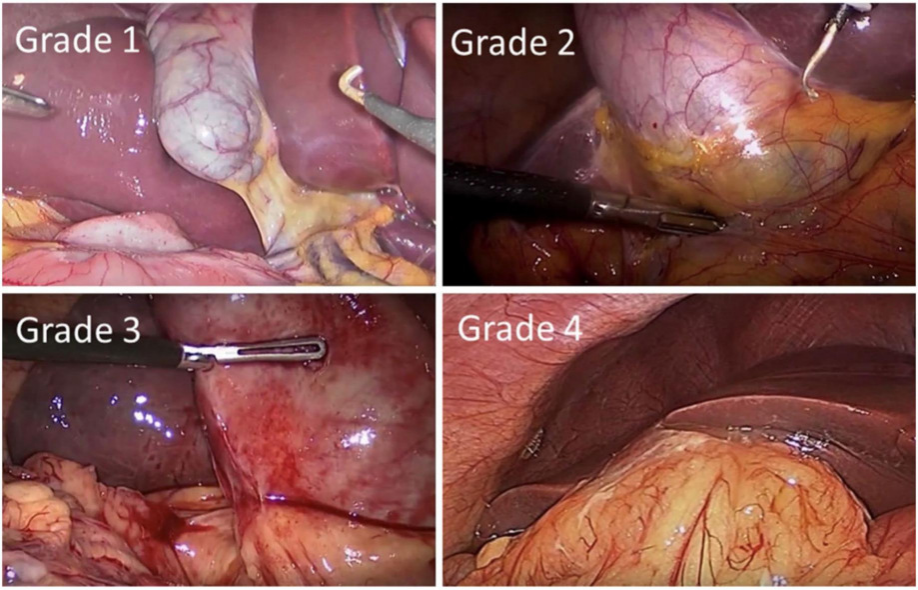
Nassar operative difficulty grades (14).

Surgeons were asked to grade the difficulty of the procedure using the Nassar difficulty grading scale (Figure 2). This scale was published in 1995 and graded operative findings from the gallbladder, cystic pedicle and associated adhesions (13). The scale is as follows: (Table 3).

**Figure 3:**
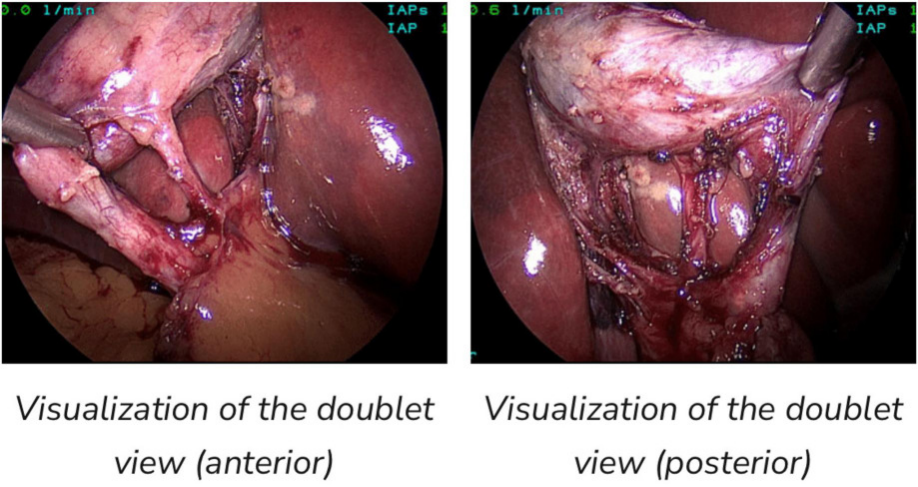
Both views fulfill criteria for CVS (15).

**Table 3:** Nassar difficulty grading scale.

Nassar difficulty grade	Gallbladder	Cystic pedicle	Adhesions
Grade I	Floppy, non-adherent	Thin and clear	Simple up to the neck/Hartmann's pouch
Grade II	Mucocele packed with stones	Fat laden	Simple up to the body
Grade III	Deep fossa, acute cholecystitis, contracted, fibrosis, Hartmans adherent to CBD, impaction	Abnormal anatomy or cystic duct—short, dilated or obscured	Dense up to fundus involving hepatic flexure or duodenum
Grade IV	Completely obscured, empyema, gangrene, mass	Impossible to clarify	Dense, fibrosis, wrapping the gallbladder, Duodenum or hepatic flexure difficult to separate

In our study, Grade 1 Nassar scale was the most commonly encountered difficulty grade (*n* = 51, 37.78%), followed by grade 2 (*n* = 25, 18.52%) (Table 4).

**Table 4:** Operative details.

Variable	Surgery performed by resident (*n* = 15)	Surgery performed by consultant (*n* = 135)
Intra-operative Nassar difficultly grading		
Scale *N* (%)		
I	8 (53.3)	51 (37.78)
II	2 (13.4)	25 (18.52)
III	0	20 (14.81)
IV	0	6 (4.44)
Unknown	5 (33.3)	33 (24.44)
Intraoperative complications *N* (%)		
Bile spillage	1 (6.7)	19 (14.1)
Stones spillage	0	5 (3.7)
Bleeding	0	5 (3.7)
Major vascular injury	0	0
Bowel injury	0	0
None	14 (93.3)	111 (82.2)
CVS achievement *N* (%)		
1. CVS not achieved	6 (40.0%)	41 (30.4)
2. CVS achieved	9 (60.0%)	94 (69.6)

### 3.4 CVS achievement rates

Of the 150 laparoscopic cholecystectomies included in the study, 135 (90.0%) were performed by consultants and 15 (10.0%) by residents. All resident-performed procedures were conducted under direct supervision, with consultants present in all cases. Total cholecystectomy was done in all patients.

Overall, the rate of CVS achievement in this study was 69.6% among consultants and 60.0% among residents. A formal time-out was done by all residents (a momentary pause to confirm achievement of CVS prior to dividing any structures), while consultants were less likely to perform a time-out. (*n* = 80, 59.3%) (Table 4).

CVS assessment relied mainly on operative notes without objective video review, introducing a potential risk of documentation bias that may limit the accuracy of these findings.

### 3.5 Primary outcome: predictive factors to achieve CVS

Binary logistic regression and chi-square analyses were conducted to examine the effect of the following variables on successful achieving the critical view of safety: Patient-related factors (age, gender, BMI, Diabetes mellitus, ASA grade, clinical frailty scale), disease-related factors (indication for surgery, Tokyo grade, history of acute cholecystitis, wall thickening of the gallbladder, pericholecystic fluid), surgeon-related factors (Level of experience, consultant subspecialty) and other peri-perative factors including interval between diagnosis and surgery, timing of surgery (emergency vs. elective) and Nassar difficulty grading scale (Table 5).

**Table 5:** Logistic regression of critical view of safety.

Coefficients	*P*-value	Odds ration	95% confidence interval
Age >= 60	0.245		
Male	0.823		
BMI >= 30	0.150		
Diabetes	0.504		
ASA grade	0.031	0.392	0.168–0.917
Clinical frailty scale	0.191		
Acute cholecystitis	0.171		
Biliary colic	0.031	0.45	0.216–0.938
Tokyo grade	0.255		
History of acute cholecystitis	0.579		
Thick-walled gallbladder	0.440		
Pericholecystic fluid	0.320		
Surgeon experience	0.558		
Consultant Subspecialty	<0.001	0.244	0.101–0.496
Days between diagnosis and surgery	0.211		
Urgency of surgery	0.01	4.013	1.319–12.211
Nassar difficulty grading scale	1		

ASA, American Society of Anesthesiologists; BMI, body mass index.

Multivariable analysis found that the following factors significantly predicted CVS achievement: ASA grade (*p* = 0.031), timing of surgery “emergency vs. elective” (*p* = 0.01), indication for surgery “acute cholecystitis vs. biliary colic” (*p* = 0.031), and consultant subspecialty “non-HPB vs. HPB” (*p* < 0.001).

In our analysis, patients who underwent emergency surgery were more likely to achieve CVS than patients who underwent elective surgery (OR: 4.013, *p* = 0.01). Similarly, patients with acute cholecystitis were more likely to have a CVS achievement than patients with biliary colic (OR: 0.45, *p* = 0.031).

Patients with ASA grade I were more likely to get CVS achieved than patients with ASA grade II (OR: 0.392, *p* = 0.031).

Non-HPB consultants had significantly higher CVS achievement rate than HPB (OR: 0.244, *p* < 0.001).

Other factors including level of surgeon experience, Tokyo severity grade, Nassar difficulty grading scale, age, male gender, BMI, diabetes mellitus and clinical frailty score were not significant.

### 3.6 Secondary outcomes: postoperative complications at 30 days

No bile duct injury was reported in this study which limits the ability to evaluate a potential association between failure to achieve CVS and the risk of BDI.

Most of the patients in this study had no complication (*n* = 132, 88%). Surgical site infection was the most common complication (*n* = 5, 3.33%) followed by postoperative pulmonary complication, intra-abdominal collection and acute pancreatitis (Table 6).

**Table 6:** 30 days postoperative outcomes.

Variable		Frequency	Percentage
Highest 30-day Clavien-Dindo	No complications	132	88.0%
	I	10	6.7%
	II	7	4.7%
	V (death)		0.7%
Unplanned critical care admission		4	2.7%
Unplanned reimaging		8	5.3%
	USS	6	4%
	CT	3	2%
	MRI	1	0.7%
	ERCP	0	0
Surgical site infection	(no complications)	145	96.7%
	I	4	2.7%
	II	1	0.7%
	V (death)	0	0.0%
Postoperative pulmonary complications	(no complications)	148	98.7%
	I	0	0.0%
	II	1	0.7%
	V (death)	1	0.7%
Bleeding	(no complications)	150	100.0%
	I	0	0.0%
	II	0	0.0%
	V (death)	0	0.0%
Intra-abdominal collection	(no complications)	148	98.7%
	I	0	0.0%
	II	2	1.3%
	V (death)	0	0.0%
Acute pancreatitis	(no complications)I	1,481	98.7%0.7%
	II	1	0.7%
	V (death)	0	0.0%
Unplanned readmission within 30 days of day of surgery		4	2.7%

A Chi-square test was conducted to examine the effect of the following variables on 30 days post operative complications:

- Failure to achieve the CVS
- Type of presentation (acute cholecystitis vs. biliary colic)
- Level of experience (consultant vs. resident)
- Intra-operative biliary spillage

All of these variables were insignificant in univariable analysis (Table 7).

**Table 7:** Chi-square test of postoperative complications.

30-day post-op complications with the following factors:	*P* value
Failure to achieve CVS	0.431
Surgeon experience	1
Type of presentation	0.370
Biliary spillage	0.265

## 4 Discussion

Strasberg's Critical View of Safety (CVS), first introduced in 1995, is widely recognized as the safest technique for identifying the elements of the Calot triangle during laparoscopic cholecystectomy (LC) (16). It aims to standardize a “mandatory” pause during LC and to make correct identification of the anatomic structures prior to cutting or clipping to minimize the risk of bile duct injury to zero (17, 18).

Despite widespread ndorsement of the CVS technique, the scientific evidence supporting it effectiveness to prevent BDI remains controversial (16), and consistent implementation of the technique varies considerably across institutions (19). Moreover, one-fourth of surgeons who reported achievement of the CVS were found to have done so inadequately (20).

Three criteria are required to achieve the CVS (21):

1. The hepatocystic triangle is cleared of fat and fibrous tissue.
2. The lower one third of the gallbladder is separated from the liver to expose the cystic plate.
3. Two and only two structures should be seen entering the gallbladder (Figure 3).

**Figure 4:**
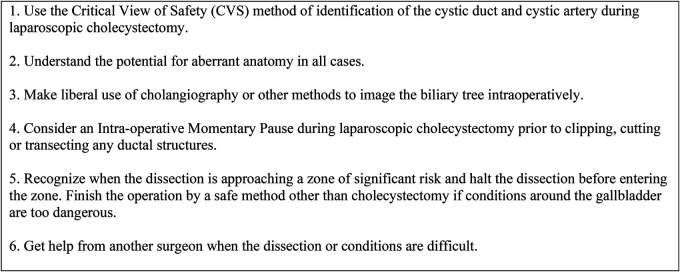
The SAGES 6-step program for safe cholecystectomy (22).

In this study, the overall rate of CVS achievement was 68.7%, which is higher than previously reported CVS success rates (23, 24).

Preoperative determination of difficult CVS can also help the surgeon in counseling the patients regarding higher chances for conversion to open procedure or bailout procedures. A prospective cohort study conducted in India found only neutrophil percent and lymphocyte percent to be independent risk factors for failure to achieve CVS (25).

While one might expect surgeon experience to be a dominant predictor, our findings suggest otherwise. Instead, physiological fitness had stronger associations with CVS achievement. In this study, patients with ASA grade I were significantly more likely to achieve CVS compared to ASA grade II, (*p* = 0.031). Which is consistent with other studies (25, 26).

Our analysis revealed that Consultants were more likely to achieve the CVS than residents (69.6% vs.60.0%) but it was not statistically significant (*p* = 0.55). While surgical expertise remains valuable, our findings imply that case complexity, individual patient challenges, and application of established protocols may exert a stronger influence on successful CVS achievement.

In this study, a formal time-out was performed by all residents prior to dividing the cystic duct to confirm the achievement of CVS, while consultants were less likely to perform a time-out. (*n* = 80, 59.3%). The SAGES 6-step program for a safe cholecystectomy (Figure 4), defines time-out as a “momentary intraoperative pause” to allow for reflection before “clipping, cutting or transecting any ductal structures” (22).

**Figure 5:**
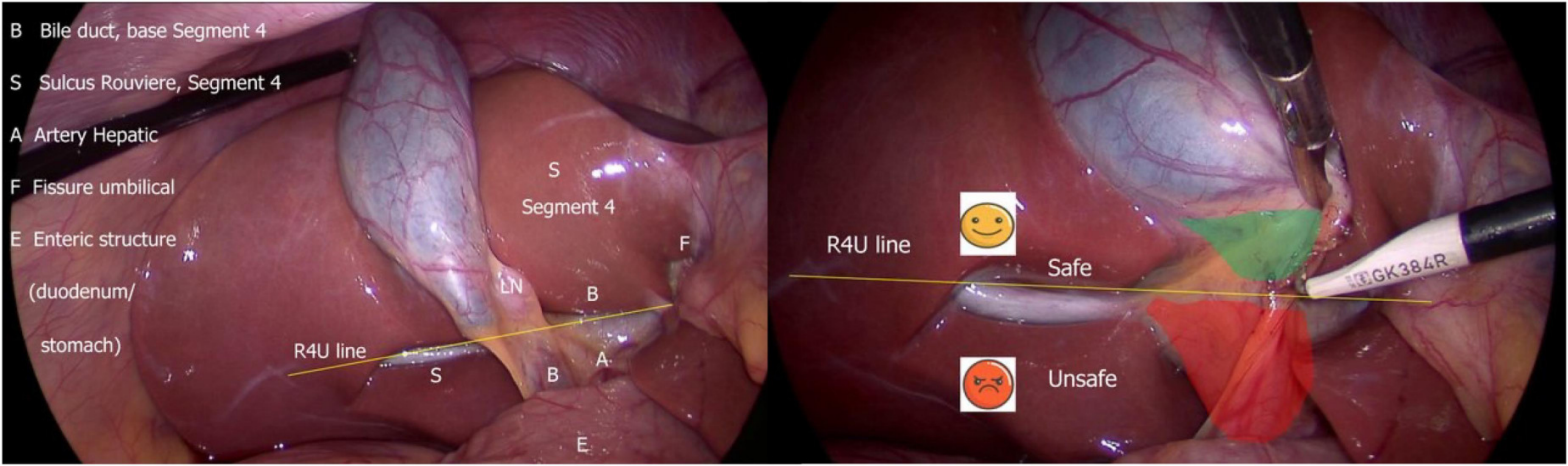
B-SAFE anatomical landmarks and R4U safety line. If Rouviere's sulcus is not present, then the imaginary line passing across the base of the segment 4 from the umbilical fissure may be extended towards right across the hepatoduodenal ligament to ascertain safe zone of dissection (27).

Using timeouts is a cognitive strategy for debiasing surgical decisions. It serves as a forcing function to shift from intuitive (fast) thinking to analytic (slow) thinking, particularly when applying the B-SAFE approach before any dissection to confirm spatial orientation (27) (Figure 5).

In our study, patients undergoing emergency cholecystectomies were more likely to have CVS achieved compared to elective cases (*p* = 0.01). 56% of patients with acute cholecystitis underwent emergent surgery (*n* = 28), whereas 98.8% of patients diagnosed with biliary colic had elective surgery (*n* = 85).

Similarly, patients with biliary colic were less likely to achieve CVS than acute cholecystitis (*p* = 0.031). This is contrary to other studies where acute cholecystitis and emergency surgery to be independent predictors of difficult LC (25, 26). While emergent cholecystectomies often present with distorted anatomy, the observed higher CVS achievement rate in acute cholecystitis cases may be explained by a heightened sense of caution and strict adherence to safety protocols in high risk settings. These findings should be interpreted cautiously, and future studies incorporating video documentation and external validation are warranted to explore these associations more robustly.

Notably, in our study, hepato-pancreato-biliary (HPB) surgeons, despite their high expertise, were paradoxically less likely to document CVS than other specialties, including surgical oncology, colorectal, upper gastrointestinal, and minimally invasive surgery (*p* < 0.001).

The paradoxical finding that non-HPB surgeons achieved CVS more frequently than HPB surgeons may reflect not only case complexity, but also variation in documentation practices, as non-HPB may follow CVS protocols more strictly or may over-document fulfillment of the three criteria to achieve the CVS—especially in protocol-driven environments like teaching hospitals. In contrast, HPB surgeons, who are often more familiar with the concept of CVS, may apply a higher threshold before declaring that the CVS has been achieved.

These findings highlight how case mix, surgical philosophy, and documentation practices can influence CVS rates. Also, they emphasize the potential role of operative culture and institutional training environments in influencing outcomes.

According to the Tokyo Guidelines 2018 for the management of acute cholecystitis, safe laparoscopic cholecystectomy is emphasized, particularly when dealing with “difficult gallbladder” cases, which may involve severe scarring or fibrosis in Calot's triangle. These guidelines recommend bailout procedures like conversion to open surgery, subtotal cholecystectomy, or the fundus-first technique to prevent bile duct-injury (28–30).

Similarly, recent “safe cholecystectomy” guidelines recommend considering bailout procedures in patients where CVS fails to be achieved (28, 31). In our study, however, all patients underwent total cholecystectomy, including those in whom the CVS could not be achieved.

Age, male gender, thickened gallbladder, pericholecystic fluid and other perioperative factors including Tokyo severity grade and Nassar difficulty grading scale were analyzed & were not statistically significant. Other studies found that age, male gender, thickened gallbladder, preoperative ERCP, to be independent predictors of difficult LC (26, 32, 33).

No significant correlation was found between CVS and 30-day postoperative complications (*p* = 0.431). This may suggest that while CVS is a preventive strategy against bile duct injury, its presence does not necessarily predict overall postoperative morbidity, which is influenced by a broader spectrum of perioperative factors.

The absence of bile duct injury in this study limits the ability to evaluate a potential association between achieving the Critical View of Safety and the risk of bile duct injury.

Importantly, our study reinforces that successful achievement of the CVS is not solely dependent on surgical experience, rather, it is a multifactorial outcome influenced by preoperative physiology, intraoperative strategy, and institutional culture.

## 5 Conclusion

Achieving the Critical View of Safety is influenced by several patient, surgical, and institutional factors. Timing of surgery (emergency, elective), indication for surgery (acute cholecystitis, biliary colic), ASA grade and surgeon subspecialty are key determinants. Surgical planning according to these factors may improve safety and standardization in laparoscopic cholecystectomy. Adherence to strict protocols is essential to ensure the consistent achievement of CVS in both elective and emergency cases. Larger multi-center studies with objective video assessments are needed to validate our findings.

### 5.1 Strengths and limitations

To the best of our knowledge, our study is among the few to prospectively assess not only patient- and disease-related variables but also surgeon characteristics as predictors for successful achievement of the CVS. Its main limitation lies in the design of the study being a single center and CVS assessment via operative notes without objective video review introducing a potential risk of documentation bias. This may have influenced findings such as the higher CVS rates among non-HPB surgeons or in emergency settings, which could reflect differences in operative note reporting rather than technical performance. Additionally, this limitation may confound interpretations related to complex anatomy and the degree of intraoperative difficulty.

The absence of bile duct injuries, while reassuring, limits our ability to assess the predictive value of CVS on this critical endpoint.

## Data Availability

The original contributions presented in the study are included in the article/Supplementary Material, further inquiries can be directed to the corresponding author.
